# An open label follow-up study on amisulpride in the add-on treatment of bipolar I patients

**DOI:** 10.1186/1745-0179-2-19

**Published:** 2006-08-24

**Authors:** Mauro Giovanni Carta, Fausta Zairo, Gisa Mellino, Maria Carolina Hardoy, Eduard Vieta

**Affiliations:** 1 Department of Public Health, University of Cagliari, Italy; 2 Bipolar Disorders Program, Clinical Institute of Neuroscience, Hospital Clinic Barcelona, IDIBAPS, University of Barcelona, Spain

## Abstract

**Background:**

Atypical antipsychotics are widely used in the treatment of bipolar disorders. Amisulpride is an atypical antipsychotic that has been proven to be effective in treatment of schizophrenia, major depressive disorder and, more recently, acute mania. At the moment, however, no study has assessed the effectiveness of this compound in maintenance therapy of bipolar disorders. The purpose of this study was to assess the long-term effectiveness of amisulpride in combination with standard treatments in patients with bipolar I disorder who have shown inadequate responses to ongoing standard therapies.

**Methods:**

The study enrolled fourteen bipolar I outpatients, not responding to ongoing standard therapy. Three patients discontinued treatment but 11 were followed-up for 11.7 ± 8.2 months before (range 3–24 months) and 5.2 ± 2.7 months after the introduction of amisulpride (range 3–9 months). Relapse rates before and during treatment with amisulpride were calculated in accordance to an increase of 1 or more in Clinical Global Impressions Scale-Bipolar Version (CGI-BP) score that was accompanied by a change in therapy or to an exacerbation of the symptoms that required hospitalization.

**Results:**

A statistically significant decrease in overall relapse rate was observed during the period of amisulpride therapy compared with months previous to the introduction of amisulpride. The relative risk of relapse in the absence of amisulpride therapy was 3.1 (χ2 = 4.2, P < 0.05). Similarly, the rates of manic/mixed and depressive relapse were decreased but only manic episodes reached statistical significance (RR = 5.3, χ2 = 5.2, P < 0.02).

**Discussion and conclusion:**

This open-label study suggests that long-term therapy with amisulpride may benefit patients by improving global symptoms of bipolar disorder and reducing the rate of manic/mixed relapses. Large, randomized, double-blind, placebo-controlled studies are needed to explore the benefits of adding long-term amisulpride to standard therapies for bipolar disorder.

## Background

Bipolar disorder is a very common mood disorder characterized by recurrent episodes of depression, mania, and/or mixed symptom states. These episodes cause unusual and extreme shifts in mood, energy, and behaviour that interfere significantly with normal, healthy functioning and strongly affects the quality of life and social functioning. Type I bipolar disorder is the most severe and usually chronic form of this disorder. The essential treatment of this affection in adults involves drugs potentially effective in controlling mania, in preventing recurrences of manic and depressive episodes and, after completing an acute phase of treatment, in maintaining a stable remission. Unfortunately longitudinal studies have proven that a large number of patients keep presenting the former symptoms, even though treated with polytherapy. Nowadays atypical antipsychotics are increasingly used as mood stabilizers during acute depressive and manic phases and are preferably used instead of the traditional ones for causing less side-effects [1]. Amisulpride is a highly tolerable atypical antipsychotic that has been reported to be effective in treatment of schizophrenia [2], major depressive disorder [3] and, more recently, acute mania [4]. At low doses, it increases dopaminergic transmission by preferentially blocking D2 and D3 presynaptic receptors; the latter property has been used in clinical trials as an additional therapy to lithium in depressed bipolar patients [5]. At the moment, however, no experimental study has assessed the effectiveness of this compound in mid-term maintenance therapy. The purpose of this study was to assess the long-term effectiveness of amisulpride in combination with standard treatments in patients with bipolar I disorder who have shown inadequate responses to ongoing standard therapies.

## Methods

The study enrolled fourteen outpatients with type I bipolar DSM-IV TR [6] disorder (8 men and 6 women, age 42.3 ± 17.8 years), not responding to ongoing standard therapy. They were treated with additional amisulpride.

Exclusion criteria included pregnancy and breastfeeding in women, hyperprolactinaemia, breast and pituitary tumors, pheochromocytoma, type Ia and III antiarrhythmics use and a recent history of alcohol or drug abuse in both men and women.

Total observation period varied from 6 to 33 months and each patient was examined at least once 3 months before and 3 months after the introduction of amisulpride as an additional therapy [11.7 ± 8.2 (Mean ± Standard Deviation) months before (range 3–24 months) and 5.2 ± 3.9 months after the introduction of amisulpride (range 3–9 months)]. Patients were examined at least once every two months and no less than eight times a year to assess response and side-effects, to record adherence to medication, and to adjust doses as necessary. Patients were rated by the same clinician throughout the study period. Patients could be withdrawn from the study at their request or on the basis of clinical judgment or because of abnormal safety assessments. Psychopathological evaluation and severity assessments were performed at baseline and at every examination. DSM-IV-TR [6] manic and depressive episodes incidence rates were measured before and after introducing amisulpride.

Relapse rates before and during treatment with amisulpride were calculated in accordance to an increase of 1 or more in Clinical Global Impressions Scale-Bipolar Version (CGI-BP) [7] score that was accompanied by a change in therapy or to an exacerbation of the symptoms who required hospitalization. Mean CGI-BP scores were calculated by comparing T-1 (3 months before the introduction of amisulpride as an additional therapy), T0 (baseline) and T1 (3 months after the introduction of amisulpride as an additional therapy).

Amisulpride was administered in combination with the outgoing therapy with a dosage ranging between 200 and 800 mg (mean 450 ± 232.9) in 10 patients with predominant manic symptoms and in 4 euthymic patients for whom it was necessary to replace the previously-used antipsychotics (N = 3 haloperidol, N = 1 risperidone) due to extrapyramidal symptoms (N = 1) or to excessive sedation (N = 3). Dosage adjustments were provided if necessary.

## Results

As 3 patients (2 women) discontinued the trial in the first two weeks because of side effects, the follow-up has been conducted on 11 patients for a duration of 11.7 ± 8.2 months before the introduction of amisulpride as additional therapy (range 3–24 months) and 5.2 ± 2.7 months after the introduction of amisulpride in therapy (range 3–9 months).

**Figure 1:**
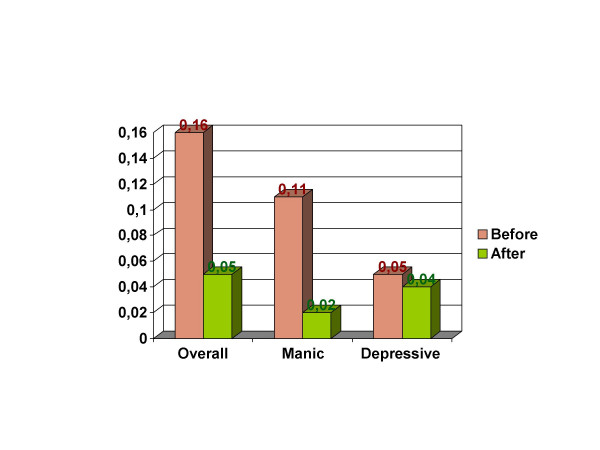
Relapse rates.

A statistically significant decrease in overall relapse rate was observed during the period of amisulpride therapy compared with months previous to the introduction of amisulpride from 21 relapse events per 129 person-months (incidence 0.16 per month) to 3 relapse events per 57 person-months (0.05) (figure 1). The relative risk of relapse in the absence of amisulpride therapy was 3.1 (χ2 = 4.2, P < 0.05).

Similarly, the rates of manic/mixed relapse and depressive relapse were decreased but only the differences of manic/mixed reached the statistical significance (figure 1). Manic or mixed relapses occurred at a rate of 15 events per 129 person-months (incidence 0.11) in the follow-up before amisulpride therapy compared with 1 event per 57 person-months (0.02) during amisulpride therapy, for a relative risk of relapse of 5.3 (χ2 = 5.2, P < 0.02).

Depressive relapses occurred at a rate of 6 events per 129 person month-years (0.05) before initiation of amisulpride therapy versus 2 events per 57 person-months (0.04) during amisulpride therapy, for a relative risk of relapse of 1.4 (χ2 = 0.22, NS). The two cases of depression, required dosage of amisulpride to be decreased to 50 mg/day.

**Figure 2:**
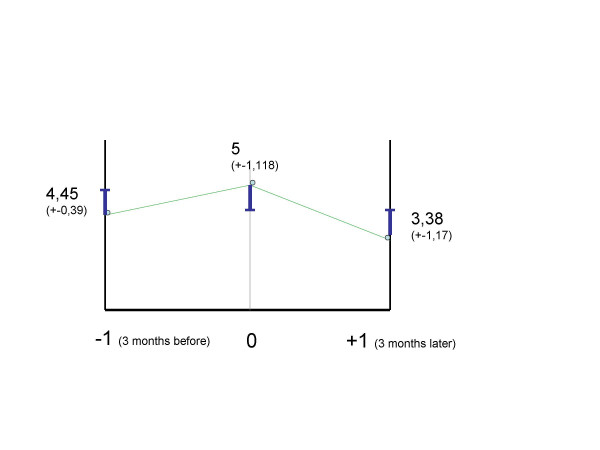
CGI-BP Score after follow up.

Improvements in mean CGI-BP score were observed comparing the mean score 3 months before (4.45 ± 0.93 [Mean ± Standard Deviation]), at baseline (5.00 ± 1.118) and 3 months after amisulpride introduction (3.82 ± 1.17, F = 3.5 (DF 2,30,32), P < 0.05; before vs baseline F = 1.47 (DF 1,20,21), P = 0.239; after vs baseline F = 5.5 (DF 1,20,21), P = 0.029 (figure 2).

Three subjects dropped out due to the side effects in the 2 weeks before the introduction of amisulpride (2 for akathisia [both female], 1 for sedation). The most frequent side effects reported during the follow-up period were mild. These side effects were: sedation (18.2%, 2 patients), weight gain (18.2%, 2 patients), and tremor (9.1%, 1 patient). There were no incidents of tardive dyskinesia. One patient discontinued amisulpride therapy after 1 year of therapy due to amenorrhea.

## Discussion

Patients with bipolar I disorder who have inadequate responses to standard pharmacological therapy remain a common problem, despite the wide array of psychotropic agents that may be utilized [8]. This open-label study suggests that long-term therapy with amisulpride may benefit patients by improving global symptoms of bipolar disorder and reducing the rate of manic/mixed relapses. In this study, treatment with amisulpride for 12–36 weeks was associated with significant improvements in relapse rates.

These results, while in agreement with a recent open prospective study that established significant improvements in manic symptoms [4], also suggest that the treatment effect observed with amisulpride is maintained in the long-term.

For each patient, long-term amisulpride treatment was added to ongoing medication, which included traditional mood stabilizers (lithium, lamotrigine, carbamazepine and oxcarbazepine) but no patients were taking divalproex or antipsychotic agents. While polypharmacy with several kinds of psychotropic therapies is common in adults and children with bipolar disorder in the community [9,10], systematic studies of long-term therapy did not evaluate amisulpride as adjunctive therapy. This study showed that adding amisulpride to a variety of ongoing stabilizers was well tolerated and associated with a reasonable low incidence of adverse events over the long term.

Some of our study data, and in particular the overcoming of depressive episodes after decreasing the amisulpride dosage, suggest that this compound may be effective as an antidepressant at low doses and as an antimanic at high doses, properties that could be used in long-term treatment of the bipolar disorder.

Although the cause is unknown, several studies have suggested an increased rate of antipsychotic-induced tardive dyskinesia and extrapyramidal symptoms (EPS) in patients with affective disorders compared with patients with schizophrenia [11,12]. Of particular importance for patients with affective disorders, no increase in tardive dyskinesia and low incidence of EPS was reported during amisulpride therapy in this study.

This study has a number of limitations, including a small sample size and the absence of a control group. Furthermore, the design of an open-label study does not include randomization techniques and blinding of investigators to patient status, which may introduce assessment bias. Nevertheless, large, randomized, double-blind, placebo-controlled studies are needed to explore the benefits of adding long-term amisulpride to standard therapies for bipolar disorder.
